# Approaches being used in the national schistosomiasis elimination programme in China: a review

**DOI:** 10.1186/s40249-017-0271-9

**Published:** 2017-03-15

**Authors:** Le-Ping Sun, Wei Wang, Qing-Biao Hong, Shi-Zhu Li, You-Sheng Liang, Hai-Tao Yang, Xiao-Nong Zhou

**Affiliations:** 1 Key Laboratory of National Health and Family Planning Commission on Parasitic Disease Control and Prevention, Wuxi, 214064 China; 2 Jiangsu Provincial Key Laboratory on Parasites and Vector Control Technology, Wuxi, 214064 China; 3 Jiangsu Institute of Parasitic Diseases, Wuxi, 214064 China; 4 School of Public Health, Fujian Medical University, Fuzhou, 350004 China; 5 National Institute of Parasitic Diseases, Chinese Center for Disease Control and Prevention, Shanghai, 200025 China; 6 Key Laboratory for Parasite and Vector Biology, National Health and Family Planning Commission, Shanghai, 200025 China; 7 WHO Collaborating Center for Tropical Diseases, National Center for International Research on Tropical Diseases, Shanghai, 200025 China

**Keywords:** Schistosomiasis japonica, *Schistosoma japonicum*, *Oncomelania hupensis*, Elimination, Snail control, Source of infection, Health education, China

## Abstract

Schistosomiasis japonica, caused by the human blood fluke *Schistosoma japonicum*, remains a major public health problem in China, although great success has been achieved. The control efforts during the past half-decade, notably the wide implementation of the new integrated strategy with emphasis on control of the source of *S. japonicum* infection across the country since 2004, has greatly reduced *S. japonicum* in humans, livestock, and intermediate host *Oncomelania hupensis* snails, and transmission control of schistosomiasis was achieved in China in 2015. A two-stage roadmap was therefore proposed for schistosomiasis elimination in 2015, with aims to achieve transmission interruption by 2020 and achieve disease elimination by 2025 in the country. During the last two decades, a variety of approaches, which target the epidemiological factors of schistosomiasis japonica have been developed, in order to block the transmission cycle of the parasite. These approaches have been employed in the national or local schistosomiasis control activities, and facilitated, at least in part, the progress of the schistosomiasis elimination programs. Here, we present an approach to control the source of *S. japonicum* infection, three new tools for snail control, three approaches for detecting and monitoring *S. japonicum* infection, and a novel model for health education. These approaches are considered to play a great role in the stage moving towards transmission interruption and elimination of schistosomiasis in China.

**Electronic supplementary material:**

The online version of this article (doi:10.1186/s40249-017-0271-9) contains supplementary material, which is available to authorized users.

## Multilingual abstracts

Please see Additional file 1 for translations of the abstract into the six official working languages of the United Nations.

## Introduction

Schistosomiasis japonica, caused by the human blood fluke *Schistosoma japonicum*, remains endemic in China, the Philippines and parts of Indonesia [1–3]. In China, the description of schistosomiasis dates back more than two millennia [4, 5]. After the founding of the People’s Republic of China, schistosomiasis was once recognized as “God of plague”, since the disease caused huge social, economic and disease burdens in the country [6]. At the initial stage of the national schistosomiasis control programme in 1950s, over 11 million people were estimated to have the disease in China [7, 8]. Then, the integrated control activities [9–15], together with strong political will and sufficient financial support [16, 17], had resulted in a remarkable decline in both the prevalence and intensity of *S. japonicum* infection [18–23].

However, there was a resurgence of schistosomiasis in China at the early 2000s [24–28], due to the termination of the World Bank Loan Project (WBLP) for Chinese Schistosomiasis Control Program [29, 30], the continuous flooding along the Yangtze River basin [31], and changes of other natural, social and economic factors [32, 33]. Since 2004, schistosomiasis has been defined as one of the top four priorities for communicable disease control by the central government [34], and a new national strategy was proposed aiming to control the transmission of *S. japonicum* in China [7]. The new strategy integrates management of the sources of *S. japonicum* infection, chemotherapy, snail control, health education, and improved sanitation and access to safe water [35–37]. The implementation of this integrated strategy has achieved great success in controlling the transmission of *S. japonicum* in the country [38–44]. By 2015, only 77.2 thousand people were estimated to have the disease in China [45], which reduced by 90.8% as compared to that in 2004 when the new integrated strategy was initiated [46], and no *S. japonicum* infection was identified in *Oncomelania hupensis* snails since 2014 [47]. Based on the control achievements, a two-stage roadmap was therefore proposed for schistosomiasis elimination in China in 2015, with aims to achieve transmission interruption by 2020 and disease elimination by 2025 [48, 49].

During the past two decades, a variety of approaches, which target the epidemiological factors of schistosomiasis japonica, have been developed, in order to block the transmission cycle of the disease. These approaches have been employed in national or local schistosomiasis control activities, and facilitated, at least in part, the progress of the schistosomiasis elimination programs. Here, we present some approaches that have shown effective to control the transmission of *S. japonicum* in China, so as to provide choice of interventions for the national schistosomiasis elimination program.

### An approach to control the source of *S. japonicum* infection

**Fig. 1:**
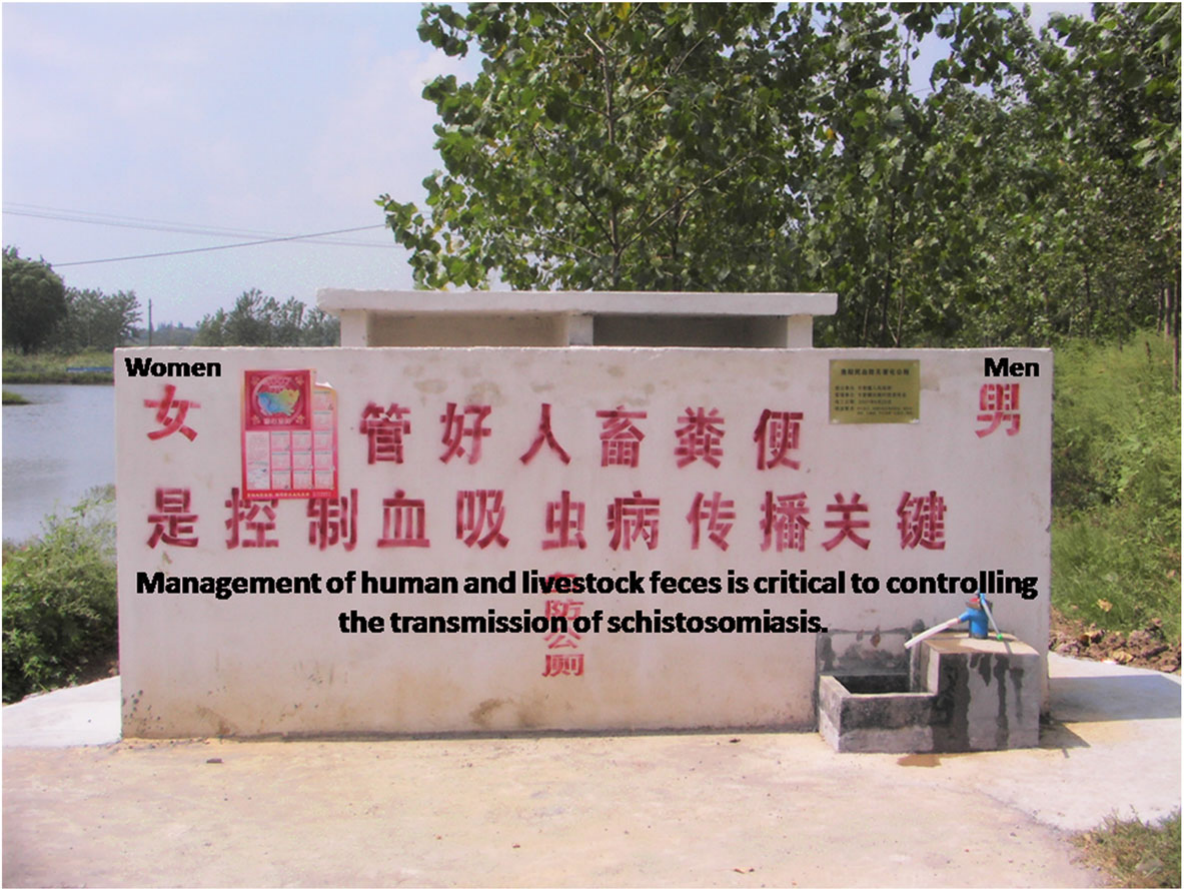
A public toilet with three-cell septic tanks built in the anchor site along the Yangtze River basin

Boatman and fisherman have a high frequency of contacting *S. japonicum*-infested water, and play a dual role in the transmission of schistosomiasis [50–52]. They act as both victims (health-harming after being infected) and transmitter of schistosomiasis (source of *S. japoncum* infection) [53–55]. Since the boatman and fishermen are characterized by frequent mobility and have relatively stable anchor sites [50–52], public toilets with three-cell septic tanks had been built on the marshlands besides the anchor sites along the Yangtze River basin (Fig. 1). The public toilets were used to collect the excrements from the boatman and fishermen, and all schistosome eggs in the night soil were killed, so as to reduce the contamination of the Yangtze River by schistosome eggs derived from the boatman and fishermen [56–58].

The public toilets have two types, 2-seat with an area of 6 m^2^ and 4-seat with an area of 13 m^2^ [56]. A total of 53 public toilets had been built in the anchor sites along the Yangtze River basin and an estimated 79.62% rate of use was observed [58]. Currently, this approach has been widely employed in the major schistosomiasis-endemic foci of China, and has become an effective tool for the management of the feces excreted from boatman and fishermen [59]. Such an approach provides a novel measure for schistosomiasis elimination in the country.

### Approaches for snail control

#### A machine simultaneously integrating mechanized environmental cleaning and automatic molluscicide treatment

**Fig. 2:**
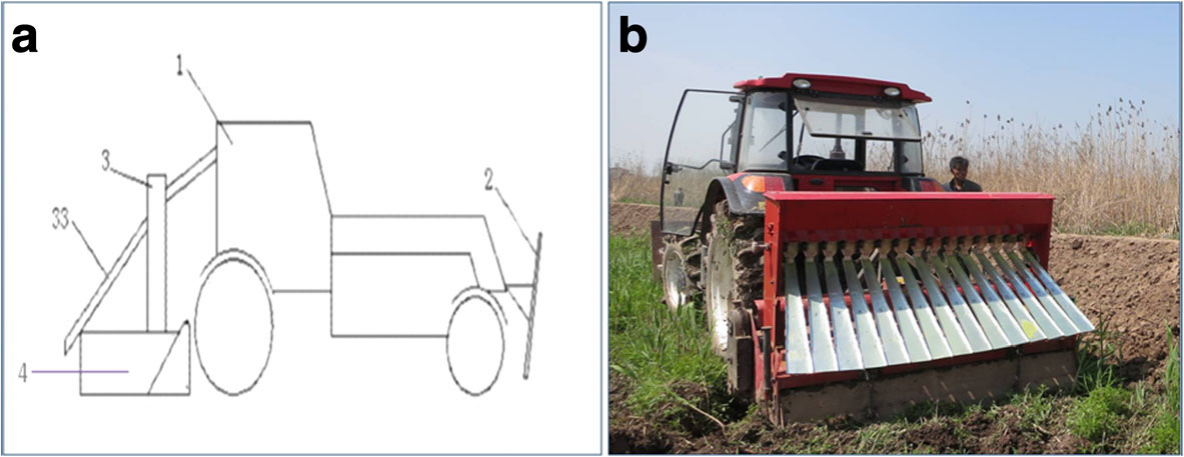
Structure diagram (**a**) and working field of the machine simultaneously integrating mechanized environmental cleaning and automatic molluscicide treatment (**b**). 1, tractor; 2, vegetation-pressing plate; 3, automatic molluscicide-releasing device; 33, comb-like molluscicide spraying retainer; 4, plough machine

Environmental vegetation is a primary factor affecting the efficiency and quality of *O. hupensis* snail control [60, 61]. A machine simultaneously integrating mechanized environmental cleaning and automatic molluscicide treatment (Fig. 2), which contains three systems including power traction, cutting up and plough, and automatic molluscicide treatment, was developed. The machine simultaneously completes the procedures of getting vegetations down and cutting vegetations into pieces, ploughing the land and molluscicide treatment with niclosamide formulations [62]. In the complicated marshland regions with vegetations, the device can complete a 3 000 m^2^ area of environmental cleaning and molluscicide treatment per working hour, and have a working efficiency similar to 56 workers; however, the economic cost is equal to approximately 1/6 human power [62]. In addition, the machine exhibit a snail control efficacy that is comparative to artificially environmental cleaning plus chemical molluscicide treatment (86.58% vs. 84.37%) [62]. It is therefore considered that this device provides a feasible tool for snail control in the large marshland regions endemic for *S. japonicum*.

#### A rapid niclosamide detector

**Fig. 3:**
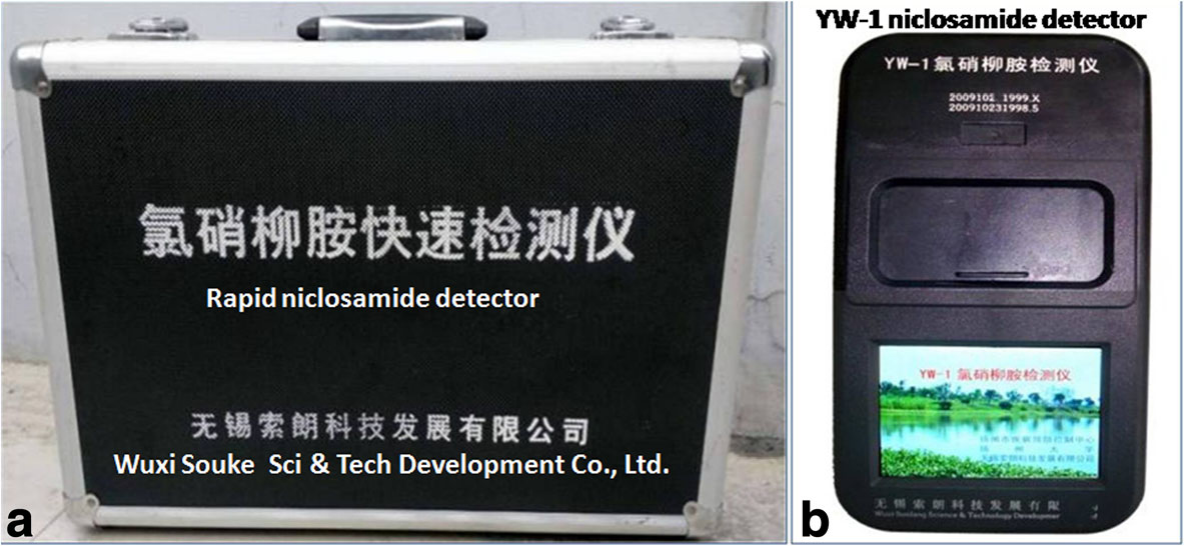
The package for the field niclosamide detector (**a**) and the field niclosamide detector (**b**)

Currently, niclosamide remains the most widely used chemical molluscicide for snail control in the endemic field of schistosomiasis worldwide [63, 64], and a real-time determination of the active niclosamide concentration is of great importance to achieve the molluscicidal efficacy and reduce the environmental toxicity [65, 66]. A niclosamide detector was developed for the rapid determination of the niclosamide concentration in the endemic field (Fig. 3). The detector has a linear range of 0 to 8 g/m^3^ and a detection limit of 0.015 g/m^3^, and it is easy to carry, which measures 2.5 cm × 9 cm × 24 cm [67]. In addition, this detector is very convenient and rapid, and has a high sensitivity for the field detection of the niclosamide concentration [67]. To date, this tool has been widely applied for the quality control of molluscicide treatment in the schistosomiasis-endemic regions of Jiangsu Province. This device is believed to provide an effective tool to support the elimination of schistosomiasis in China.

#### Snail control with black plastic film coverage

**Fig. 4:**
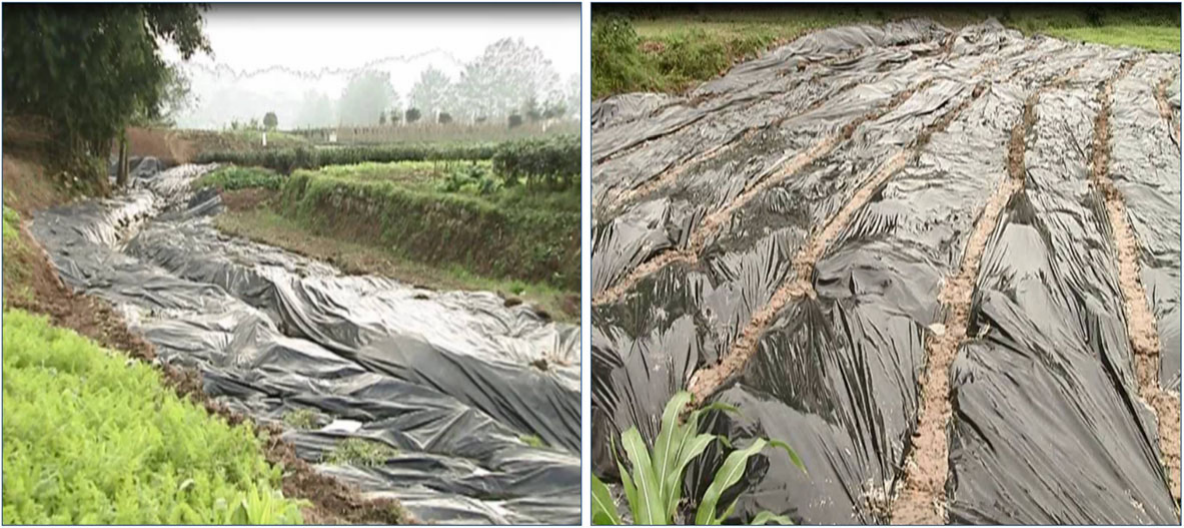
Control of the intermediate host snails through coverage with the black plastic film

The approximate temperature for snail breeding and reproduction is 15 to 25 °C; snails cannot survive at > 29 °C, and may die within several hours at > 40 °C [61]. To achieve snail control in mountainous and hilly regions, a snail control approach with black plastic film coverage has been developed (Fig. 4). In the mountainous regions, the density of living snails reduced by 67.71%, 93.06% and 100% after 7, 10 and 30 days of coverage with the black plastic film [68], and in the marshland and lake regions, the density of living snails reduced by 20.77% and 96.92% following 15 and 30 days of coverage with the black plastic film, respectively [69]. More importantly, the plastic film coverage is nontoxic to aquacultures and is active against snails and snail eggs in the soil layer, which is effective to inhibit the reproduction and breeding of the offspring snails [70]. This snail control approach is applicable for the snail control in specific snail habitats, such as fish ponds [71]. Such a method was employed as the major snail control measures for achieving the elimination of schistosomiasis in Sichuan Province in 2015, where mountainous and hilly regions are the predominant endemic areas [72]. Currently, this approach is recommended by the Ministry of Health, China, as an effective snail control intervention in the marshland and lake, and plain regions of China during the stage moving towards elimination of schistosomiasis.

### Tools for detection and monitoring of *S. japonicum* infection

#### An intelligent device for detecting S. japonicum-infested water

**Fig. 5:**
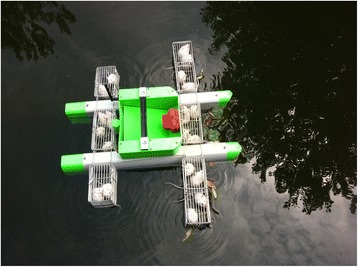
An intelligent device for detecting *S. japonicum*-infested water with sentinel mice

According to the biological feature of *S. japonicum* cercariae that float on water surface and cannot migrate actively [73], an intelligent device was developed for detecting *S. japonicum*-infested water with a mouse bioassay (Fig. 5). This device increases the likelihood of detection of *S. japonicum* cercariae through the remote-controlled movement in the water body. Field test showed that the detector reduced the detection from 8 h to 1 h, and increased *S. japonicum* infection from 15 to 40% in sentinel mice, and the intensity of infection (worm burden) from 0.25 to 2.55 worms per mouse [74]. The intelligent detector greatly enhances the efficiency for the field detection of the infested water, and has played a critical role in the surveillance-response system for schistosomiasis along the lower reaches of the Yangtze River basin [75].

#### A kit for detecting S. japonicum DNA in O. hupensis snails

**Fig. 6:**
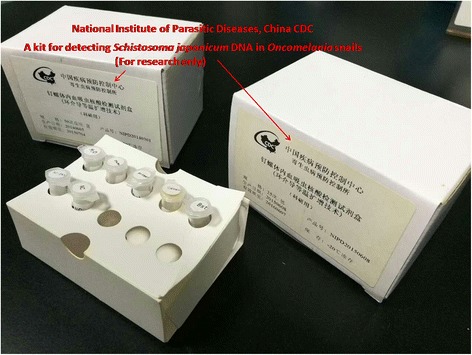
A kit for detecting genomic *S. japonicum* DNA in *O. hupensis* snails

To achieve early detection of *S. japonicum* in snails, a rapid extraction of snail genomic DNA combined with loop-mediated isothermal amplification (LAMP) assay was developed [76], which greatly reduces the identification of infected snails from 60 days (dissection of snails) to approximately one week [77]. As compared to currently available commercial imported reagents, this kit (Fig. 6) has comparable detection efficiency, but showing an over 50% reduction in cost [77], which has been integrated in the national schistosomiasis control programs of China. During the process towards transmission interruption and elimination of schistosomiasis, this assay, which greatly improves the sensitivity for detection of *S. japonicum* infection in snails in relative to conventional microscopic approaches, may provide an effective approach for the rapid identification and timely elimination of the risk of schistosomiasis transmission [78, 79].

#### Web- and Google Earth-based surveillance-response system

**Fig. 7:**
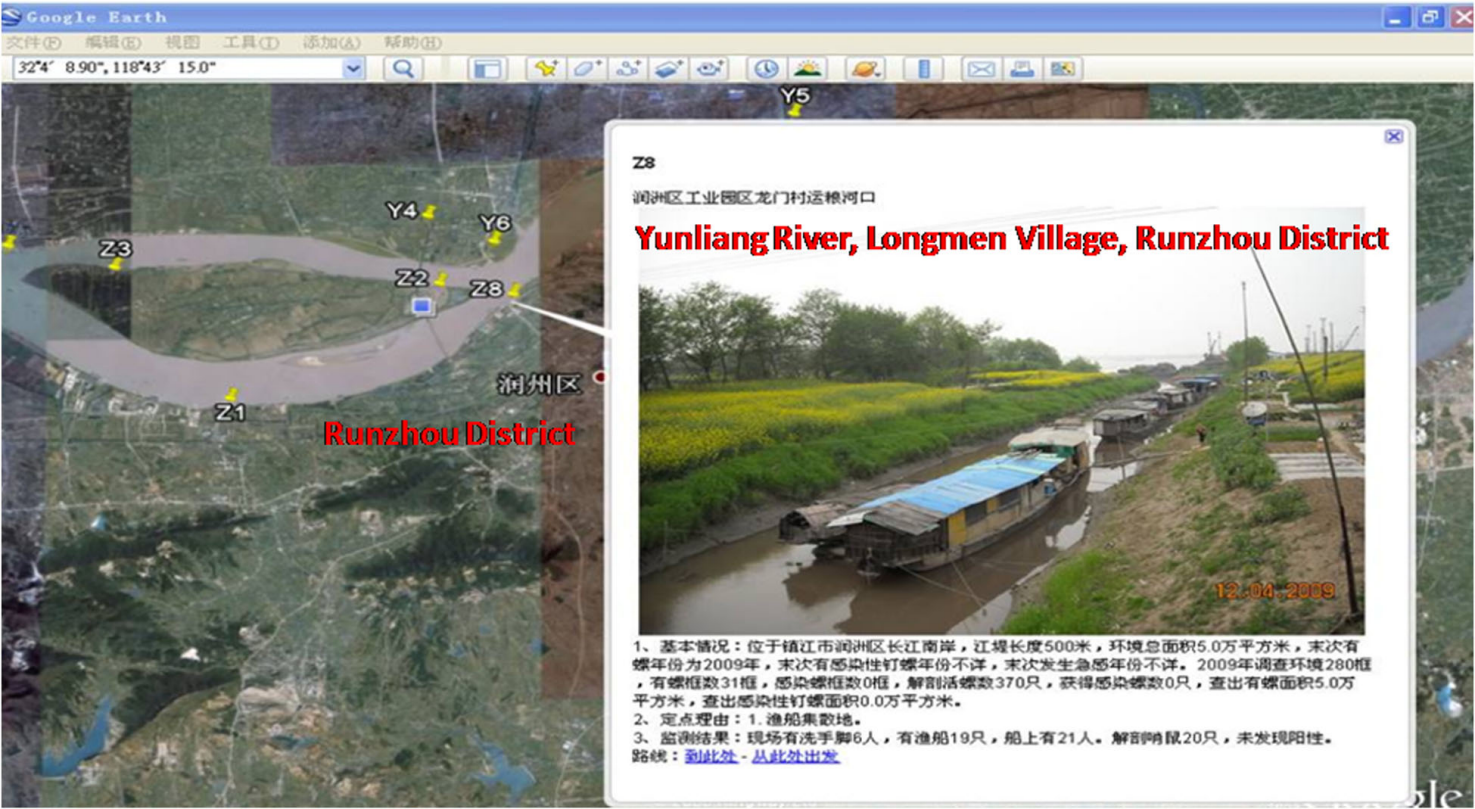
Web- and Google Earth-based surveillance-response system

In the field schistosomiasis control, the rapid release and sharing of the monitoring information is the prerequisite to the rapid response [80]. A surveillance-response system of schistosomiasis was developed based on Web and Google Earth (Fig. 7), which effectively enhances the use of the monitoring information and achieves the synchronous visualization of the monitoring information [81, 82]. This system displays the graphs and texts directly and clearly, and is easy and simple to perform [83], which plays a critical role in the elimination of schistosomiasis in China, notably in the stage moving towards schistosomiasis elimination.

### A novel model for health education

As described above, boatman and fisherman have a high likelihood and a high prevalence of *S. japonicum* infection [50–52], and they are recognized as the key target population of health education for schistosomiasis control [53]. However, the health education materials are usually not reached to the boatman and fisherman due to their high motility. A new model was therefore developed of schistosomiasis control health education. Firstly, a group of active, respected boatman and fishermen with high education levels are selected as health education volunteers. Then, the volunteers receive training pertaining to schistosomiasis prevention and control by professional staff from local CDC, and the volunteers are ensured to seize the techniques of health education regarding schistosomiasis prevention and control knowledge. Subsequently, the volunteers transmit the schistosomiasis prevention and control knowledge to the massive boatman and fishermen (one volunteer is responsible for boatmen and fishermen living in 10 boats), and participate in the health education interventions targeting the boatman and fishermen. In addition, the volunteers help professional staff to alter the incorrect behaviors, remind the implementation of self-protective measures and prohibit the pouring of the contaminated feces into water. They also help to record the use of feces container in boats and the use of harmless public toilets at the anchor sites. To evaluate the effectiveness of this health education model on schistosomiasis control, a questionnaire survey was conducted among the boatmen and fishermen. The results showed that the 3-year (2005 to 2007) implementation of this health education model increased the awareness of schistosomiasis control knowledge from 23.85 to 95.7% and percentage of correct schistosomiasis control behavior from 6.59 to 53.42%, the use of public toilets from 0 to 80.21% and the use of on-boat fecal container from 0 to 54.52%, respectively, and the sero-prevalence of *S. japonicum* infection decreased from 27.95% in 2004 to 19.24% in 2005, 12.27% in 2006 and 8.15% in 2007, respectively [84]. The results demonstrate that this new health education model improves the awareness of schistosomiasis prevention and control knowledge and may correct the incorrect health behaviors, which play an active role in the prevention and control of schistosomiasis among the boatman and fishermen.

## Conclusions

In this study, we present several approaches that had been developed during the past 2 decades, and they have been proved to effectively facilitate the progress towards the elimination of schistosomiasis in China. Actually, there are many other diagnostics, therapeutics, information, education and communication (IEC) materials, and snail control interventions developed in China, which are not presented in this review. Further systematic reviews to describe the role of all approaches in the national schistosomisis elimination program of China are required.

With the agenda set for global schistosomiasis elimination [85], Africa, the most severely afflicted regions due to schistosomiasis [86, 87], is also striving to eliminate this neglected tropical disease [88]. However, it is almost impossible to achieve schistosomiasis elimination depending on mass drug administration (MDA) with praziquantel alone, which remains the primary strategy for schistosomiasis control until now [89–91]. Currently, China is aiding the elimination of schistosomiasis from mainland Africa [92, 93]. The approaches, which have been proved to be effective to block the transmission cycle of *S. japonicum* in China, may be effective to control the transmission of other *Schistosoma* species, with adaption to local epidemiological profiles. Further studies to assess the feasibility and effectiveness of these approaches in regions endemic for *S. mansoni* and *S. haematobium* seem justified.

## Additional file

Additional file 1: Multilingual abstracts in the six official working languages of the United Nations. (PDF 603 kb)

## Abbreviations

CDC: Center for disease control and prevention
IEC: Information, education and communication
LAMP: Loop-mediated isothermal amplification
MDA: Mass drug administration
WBLP: World bank loan project.

## References

[1] Chen MG (2014). Assessment of morbidity due to *Schistosoma japonicum* infection in China. Infect Dis Poverty..

[2] Zhou XN, Bergquist R, Leonardo L, Yang GJ, Yang K, Sudomo M, Olveda R (2010). Schistosomiasis japonica control and research needs. Adv Parasitol..

[3] Bergquist R, Tanner M (2010). Controlling schistosomiasis in Southeast Asia: a tale of two countries. Adv Parasitol..

[4] Zhou XN, Wang LY, Chen MG, Wu XH, Jiang QW, Chen XY (2005). The public health significance and control of schistosomiasis in China--then and now. Acta Trop..

[5] Utzinger J, Zhou XN, Chen MG, Bergquist R (2005). Conquering schistosomiasis in China: the long march. Acta Trop..

[6] Yuan HC (1995). Schistosomiasis control in China. Mem Inst Oswaldo Cruz..

[7] Wang LD, Chen HG, Guo JG, Zeng XJ, Hong XL, Xiong JJ (2009). A strategy to control transmission of *Schistosoma japonicum* in China. N Engl J Med..

[8] Collins C, Xu J, Tang S (2012). Schistosomiasis control and the health system in P.R. China. Infect Dis Poverty.

[9] Xu J, Steinman P, Maybe D, Zhou XN, Lv S, Li SZ (2016). Evolution of the national schistosomiasis control programmes in the People's Republic of China. Adv Parasitol..

[10] Zhang SQ, Sun CS, Wang M, Lin DD, Zhou XN, Wang TP (2016). Epidemiological features and effectiveness of schistosomiasis control programme in lake and marshland region in the People's Republic of China. Adv Parasitol..

[11] Liu Y, Zhou YB, Li RZ, Wan JJ, Yang Y, Qiu DC (2016). Epidemiological features and effectiveness of schistosomiasis control programme in mountainous and hilly region of the People's Republic of China. Adv Parasitol..

[12] Shi L, Li W, Wu F, Zhang JF, Yang K, Zhou XN (2016). Epidemiological features and control progress of schistosomiasis in waterway-network region in the People's Republic of China. Adv Parasitol..

[13] Yang Y, Zhou YB, Song XX, Li SZ, Zhong B, Wang TP (2016). Integrated control strategy of schistosomiasis in the People's Republic of China: projects involving agriculture, water conservancy, forestry, sanitation and environmental modification. Adv Parasitol..

[14] Cao ZG, Zhao YE, Lee Willingham A, Wang TP (2016). Towards the elimination of schistosomiasis japonica through control of the disease in domestic animals in the People's Republic of China: a tale of over 60 years. Adv Parasitol..

[15] Chen L, Zhong B, Xu J, Li RZ, Cao CL (2016). Health education as an important component in the national schistosomiasis control programme in the People's Republic of China. Adv Parasitol..

[16] Wang W, Dai JR, Liang YS (2014). Apropos: factors impacting on progress towards elimination of transmission of schistosomiasis japonica in China. Parasit Vectors..

[17] Zhu H, Yap P, Utzinger J, Jia TW, Li SZ, Huang XB, Cai SX (2016). Policy support and resources mobilization for the national schistosomiasis control programme in the People's Republic of China. Adv Parasitol..

[18] Wang JL, Li TT, Huang SY, Cong W, Zhu XQ (2016). Major parasitic diseases of poverty in mainland China: perspectives for better control. Infect Dis Poverty..

[19] Liu L, Yang GJ, Zhu HR, Yang K, Ai L (2014). Knowledge of, attitudes towards, and practice relating to schistosomiasis in two subtypes of a mountainous region of the People's Republic of China. Infect Dis Poverty..

[20] Zhou YB, Liang S, Chen Y, Jiang QW (2016). The Three Gorges Dam: Does it accelerate or delay the progress towards eliminating transmission of schistosomiasis in China?. Infect Dis Poverty..

[21] Zheng Q, Vanderslott S, Jiang B, Xu LL, Liu CS, Huo LL (2013). Research gaps for three main tropical diseases in the People's Republic of China. Infect Dis Poverty..

[22] Shin JW, Chen JX, Zhang DH, Lin WC, Shen B, Ji MJ (2014). Cross-strait parasitological research priorities arrived at by historical tracking and advanced dialogue. Infect Dis Poverty..

[23] Zhou XN, Guo JG, Wu XH, Jiang QW, Zheng J, Dang H (2007). Epidemiology of schistosomiasis in the People's Republic of China, 2004. Emerg Infect Dis..

[24] Liang S, Yang C, Zhong B, Qiu D (2006). Re-emerging schistosomiasis in hilly and mountainous areas of Sichuan, China. Bull World Health Organ.

[25] Wang Q, Xu J, Zhang LJ, Zheng H, Ruan Y, Hao YW (2015). Analysis of endemic changes of schistosomiasis in China from 2002 to 2010. Chin J Schisto Control..

[26] Chen XY, Wu XH, Wang LY, Dang H, Wang Q, Zheng J (2003). Schistosomiasis situation in the People’s Republic of China in 2002. Chin J Schisto Control..

[27] Xiao DL, Yu Q, Dang H, Guo JG, Zhou XN, Wang LY (2004). Schistosomiasis situation in the People’s Republic of China in 2003. Chin J Schisto Control..

[28] Hao Y, Wu XH, Xia G, Zheng H, Guo JG, Wang LY (2005). Schistosomiasis situation in the People’s Republic of China in 2004. Chin J Schisto Control..

[29] Xianyi C, Liying W, Jiming C, Xiaonong Z, Jiang Z, Jiagang G, Xiaohua W, Engels D, Minggang C (2005). Schistosomiasis control in China: the impact of a 10-year World Bank Loan Project (1992–2001). Bull World Health Organ..

[30] Hu Y, Xiong C, Zhang Z, Luo C, Ward M, Gao J (2014). Dynamics of spatial clustering of schistosomiasis in the Yangtze River Valley at the end of and following the World Bank Loan Project. Parasitol Int..

[31] Wu XH, Zhang SQ, Xu XJ, Huang YX, Steinmann P, Utzinger J (2008). Effect of floods on the transmission of schistosomiasis in the Yangtze River valley, People's Republic of China. Parasitol Int.

[32] Zhou YB, Liang S, Jiang QW (2012). Factors impacting on progress towards elimination of transmission of schistosomiasis japonica in China. Parasit Vectors..

[33] Yang GJ, Utzinger J, Zhou XN (2015). Interplay between environment, agriculture and infectious diseases of poverty: case studies in China. Acta Trop..

[34] Wang L, Utzinger J, Zhou XN (2008). Schistosomiasis control: experiences and lessons from China. Lancet..

[35] Wang LD, Guo JG, Wu XH, Chen HG, Wang TP, Zhu SP (2009). China's new strategy to block *Schistosoma japonicum* transmission: experiences and impact beyond schistosomiasis. Trop Med Int Health..

[36] Seto EY, Remais JV, Carlton EJ, Wang S, Liang S, Brindley PJ (2011). Toward sustainable and comprehensive control of schistosomiasis in China: lessons from Sichuan. PLoS Negl Trop Dis..

[37] Zhou XN (2012). Prioritizing research for "One health - One world". Infect Dis Poverty..

[38] Chen YY, Liu JB, Huang XB, Cai SX, Su ZM, Zhong R (2014). New integrated strategy emphasizing infection source control to curb Schistosomiasis japonica in a marshland area of Hubei Province, China: findings from an eight-year longitudinal survey. PLoS One.

[39] Zhou YB, Liang S, Chen GX, Rea C, Han SM, He ZG (2013). Spatial-temporal variations of *Schistosoma japonicum* distribution after an integrated national control strategy: a cohort in a marshland area of China. BMC Public Health..

[40] Hong XC, Xu XJ, Chen X, Li YS, Yu CH, Yuan Y (2013). Assessing the effect of an integrated control strategy for schistosomiasis japonica emphasizing bovines in a marshland area of Hubei Province, China: a cluster randomized trial. PLoS Negl Trop Dis.

[41] Liu R, Dong HF, Jiang MS (2013). The new national integrated strategy emphasizing infection sources control for schistosomiasis control in China has made remarkable achievements. Parasitol Res..

[42] Sun LP, Wang W, Liang YS, Tian ZX, Hong QB, Yang K (2011). Effect of an integrated control strategy for schistosomiasis japonica in the lower reaches of the Yangtze River, China: an evaluation from 2005 to 2008. Parasit Vectors..

[43] Zhou YB, Liang S, Chen GX, Rea C, He ZG, Zhang ZJ (2011). An integrated strategy for transmission control of *Schistosoma japonicum* in a marshland area of China: findings from a five-year longitudinal survey and mathematical modeling. Am J Trop Med Hyg..

[44] Hong QB, Yang K, Huang YX, Sun LP, Yang GJ, Gao Y (2011). Effectiveness of a comprehensive schistosomiasis japonica control program in Jiangsu province, China, from 2005 to 2008. Acta Trop..

[45] Zhang LJ, Xu ZM, Qian YJ, Dang H, Lu S, Xu J (2016). Endemic situation of schistosomiasis in the People’s Republic of China in 2015. Chin J Schisto Control..

[46] Li RM, Fu HS. Evaluation of endemic situation and control strategy of schistosomiasis in Gaochun County. Chin J Schisto Control. 2007;19:468–70 (in Chinese).

[47] Lei ZL, Zhang LJ, Xu ZM, Dang H, Xu J, Lv S (2015). Endemic status of schistosomiasis in the People’s Republic of China in 2014. Chin J Schisto Control..

[48] Lei ZL, Zhou XN (2015). Eradication of schistosomiasis: a new target and a new task for the National Schistosomiasis Control Porgramme in the People's Republic of China. Chin J Schisto Control..

[49] Zhou XN (2016). Implementation of precision control to achieve the goal of schistosomiasis elimination in China. Chin J Schisto Control..

[50] Li YS, Yu DB (1991). *Schistosoma japonicum* infection among migrant fishermen in the Dongting Lake region of China. Trans R Soc Trop Med Hyg..

[51] Ross AG, Yuesheng L, Sleigh AS, Yi L, Williams GM, Wu WZ (1997). Epidemiologic features of *Schistosoma japonicum* among fishermen and other occupational groups in the Dongting Lake region (Hunan Province) of China. Am J Trop Med Hyg..

[52] Li YS, He YK, Zeng QR, McManus DP (2003). Epidemiological and morbidity assessment of *Schistosoma japonicum* infection in a migrant fisherman community, the Dongting Lake region, China. Trans R Soc Trop Med Hyg..

[53] Ding GS (2012). Surveillance on schistosomiasis of boat fishermen along Yangtze River in Nantong City from 2006 to 2010. Chin J Schisto Control..

[54] Cao CL, Bao ZP, Zhu HQ, Yu Q, Li SZ, Wang Q (2010). Analysis of schistosomiasis control requirements in boat fishermen in lake regions. Chin J Schisto Control..

[55] Qian YJ, Li SZ, Xu J, Yang K, Huang YX, Cao ZG (2015). Potential schistosomiasis foci in China: a prospective study for schistosomiasis surveillance and response. Acta Trop..

[56] Gao Y, Yang J, Sun LP, Huang YX, Xue ZQ, Ma YC (2008). Study on schistosomiasis control measures in mobile boat fishermen I Harmless public toilets at fixed anchor points. Chin J Schisto Control..

[57] Gao Y, Sun LP, Wu HH, Yang J, Hong QB, Tian ZX (2009). Study on schistosomiasis control measures in mobile boat fishermen II Effect of comprehensive measures with emphasis on management of boat fishermen feces for schistosomiasis control. Chin J Schisto Control..

[58] Gao Y, Sun LP, Zuo YP, Xu YH, Zhang ZQ, Ma YC (2011). Study on schistosomiasis control measures in mobile boat fishermen III Construction and application of regional joint mechanism for schistosomiasis examination and treatment in boatmen. Chin J Schisto Control..

[59] Dang H, Xu J, Li SZ, Cao ZG, Huang YX, Wu CG (2014). Monitoring the transmission of *Schistosoma japonicum* in potential risk regions of China, 2008–2012. Int J Environ Res Public Health..

[60] Ohmae H, Iwanaga Y, Nara T, Matsuda H, Yasuraoka K (2003). Biological characteristics and control of intermediate snail host of *Schistosoma japonicum*. Parasitol Int..

[61] Li ZJ, Ge J, Dai JR, Wen LY, Lin DD, Madsen H (2016). Biology and control of snail intermediate host of *Schistosoma japonicum* in the People's Republic of China. Adv Parasitol..

[62] Wang FB, Ma YC, Sun LP, Hong QB, Gao Y, Zhang CL (2016). Integration and demonstration of key techniques in surveillance and fore-cast of schistosomiasis in Jiangsu Province III Development of a machine simultaneously integrating mechanized environmental cleaning and automatic mollusciciding. Chin J Schisto Control..

[63] Dai JR, Li YZ, Wang W, Xing YT, Qu GL, Liang YS (2015). Resistance to niclosamide in *Oncomelania hupensis*, the intermediate host of *Schistosoma japonicum*: should we be worried?. Parasitology..

[64] Sokolow SH, Wood CL, Jones IJ, Swartz SJ, Lopez M, Hsieh MH (2016). Global assessment of schistosomiasis control over the past century shows targeting the snail intermediate host works best. PLoS Negl Trop Dis..

[65] Gu XG (2004). Main problems in application of niclosamide in field. Chin J Schisto Control..

[66] Lu DB (2004). Some problems in application of niclosamide. Chin J Schisto Control..

[67] Jiang YF, Wang J, Ji ZP, Xu ZJ, Gao Y, Shao AM (2009). Development of method and detector for niclosamide detection in field. Chin J Schisto Control..

[68] Zhu HQ, Zhang GR, Zhong B, Tang SG, Cao CL, Jia B (2011). Molluscicidal effect of film on ditches in mountainous schistosomiasis endemic regions. Chin J Schisto Control..

[69] Zheng SB, Zhou YB, Li LH, Wu JY, Yao BD, Zhu SP (2013). Short-term effect of black film covering on *Oncomelania hupensis* snail control in marshland and lake regions. Chin J Schisto Control.

[70] Liu HC, Zhong CH, Wan GQ, Cheng B, Wang YX, Tian RL (2013). Molluscicidal effect of plastic film mulch covering method. Chin J Schisto Control..

[71] Zhu HQ, Zhang GR, Zhong B, Tang SG, Cao CL, Jia B (2011). Toxicity of niclosamide with plastic film mulching to fish. Chin J Schisto Control..

[72] Wan JJ, Xu L, Wu ZS, Xu J, Chen J, Chen L (2016). Schistosomiasis control progress and endemic situation in Sichuan Province. Chin J Schisto Control..

[73] Haas W, Granzer M, Garcia EG (1987). Host identification by *Schistosoma japonicum* cercariae. J Parasitol..

[74] Yang K, Sun LP, Liang YS, Wu F, Li W, Zhang JF (2013). *Schistosoma japonicum* risk in Jiangsu province, People's Republic of China: identification of a spatio-temporal risk pattern along the Yangtze River. Geospat Health..

[75] Liang YS, Huang YX, Hong QB, Yang K, Sun LP, Dai JR (2012). Novel strategies and technologies to achieve the transmission control of schistosomiasis in Jiangsu Province. Chin J Schisto Control..

[76] Chen JH, Wen LY, Zhang XZ, Zhang JF, Yu LL, Hong LD (2006). Development of a PCR assay for detecting *Schistosoma japonicum*-Infected *Oncomelania hupensis*. Chin J Parasitol Parasit Dis..

[77] Xiong CR, Yin XR, Song LJ, Shen S, Wang J, Gao H (2014). Comparison of the effectiveness of loop-mediated isothermal DNA amplification (LAMP) and microscopic dissection at detecting snails infected with *Schistosoma japonicum*. Chin J Pathogen Biol..

[78] Tambo E, Ai L, Zhou X, Chen JH, Hu W, Bergquist R (2014). Surveillance-response systems: the key to elimination of tropical diseases. Infect Dis Poverty..

[79] Zhou XN, Bergquist R, Tanner M (2013). Elimination of tropical disease through surveillance and response. Infect Dis Poverty..

[80] Bergquist R, Yang GJ, Knopp S, Utzinger J, Tanner M (2015). Surveillance and response: Tools and approaches for the elimination stage of neglected tropical diseases. Acta Trop..

[81] Sun LP, Liang YS, Wu HH, Tian ZX, Dai JR, Yang K (2011). A Google Earth-based surveillance system for schistosomiasis japonica implemented in the lower reaches of the Yangtze River, China. Parasit Vectors.

[82] Yang K, Sun LP, Huang YX, Yang GJ, Wu F, Hang DR (2012). A real-time platform for monitoring schistosomiasis transmission supported by Google Earth and a web-based geographical information system. Geospat Health..

[83] Sun LP, Liang YS, Tian ZX, Dai JR, Hong QB, Gao Y (2009). Surveillance and forecast system of schistosomiasis in Jiangsu Province II Establishment of real-time operation and expression platform based on Google Earth. Chin J Schisto Control..

[84] Liu XL, Ma YC, Wang FB (2009). Effect of health education on schistosomiasis control in fishermen and boatmen. Chin J Schisto Control..

[85] Rollinson D, Knopp S, Levitz S, Stothard JR, Tchuem Tchuenté LA, Garba A (2013). Time to set the agenda for schistosomiasis elimination. Acta Trop..

[86] Colley DG, Bustinduy AL, Secor WE, King CH (2014). Human schistosomiasis. Lancet..

[87] Lai YS, Biedermann P, Ekpo UF, Garba A, Mathieu E, Midzi N (2015). Spatial distribution of schistosomiasis and treatment needs in sub-Saharan Africa: a systematic review and geostatistical analysis. Lancet Infect Dis..

[88] Stothard JR, Campbell SJ, Osei-Atweneboana MY, Durant T, Stanton MC, Biritwum NK (2017). Towards interruption of schistosomiasis transmission in sub-Saharan Africa: developing an appropriate environmental surveillance framework to guide and to support 'end game' interventions. Infect Dis Poverty..

[89] Olveda DU, McManus DP, Ross AG (2016). Mass drug administration and the global control of schistosomiasis: successes, limitations and clinical outcomes. Curr Opin Infect Dis..

[90] Secor WE. Early lessons from schistosomiasis mass drug administration programs. F1000Res. 2015;4. Faculty Rev-1157. 10.12688/f1000research.6826.1 PMC4752026 26937275

[91] Ross AG, Olveda RM, Li Y (2015). An audacious goal: the elimination of schistosomiasis in our lifetime through mass drug administration. Lancet..

[92] Xu J, Bergquist R, Qian YJ, Wang Q, Yu Q, Peeling R (2016). China-Africa and China-Asia collaboration on schistosomiasis control: a SWOT analysis. Adv Parasitol..

[93] Xu J, Yu Q, Tchuenté LA, Bergquist R, Sacko M, Utzinger J (2016). Enhancing collaboration between China and African countries for schistosomiasis control. Lancet Infect Dis..

