# Supplementary material for: Approaches being used in the national schistosomiasis elimination programme in China: a review
Source: Infect Dis Poverty. 2017 Mar 15;6:55. doi: 10.1186/s40249-017-0271-9 (PMC5351197; doi:10.1186/s40249-017-0271-9)
Supplement: Additional file 1: — Multilingual abstracts in the six official working languages of the United Nations. (PDF 603 kb) [file 40249_2017_271_MOESM1_ESM.pdf]

Translation of the abstract into the six official working languages of the United Nations

مقاربات مستخدمة في البرنامج الوطني للقضاء على البلهارسيا في الصين: مراجعة

لو بينغ صن، وي وانغ، كينغ بياو هونغ، شي زو لي، يو شانغ ليانغ، هاي تاو يانغ، شياو نونغ زو

خلاصة

الجابونيكا البلهارسيا الناجمة عن طفيليات الدم البشري البلهارسية اليابانية، لا تزال تمثل مشكلة صحية عامة ورئيسية في الصين، على الرغم من النجاح الكبير الذي تم تحقيقه. جهود السيطرة التي بذلت خلال نصف العقد الماضي، ولا سيما في تنفيذ الاستراتيجية الواسعة والجديدة والمتكاملة مع التشديد على السيطرة على مصدر البلهارسيات اليابانية العدوى في جميع أنحاء البلاد منذ عام 2004، وقد تقلص إلى حد كبير البلهارسيات اليابانية في البشر والماشية، ومضيف وسيط قواقع الماء العذب من فصيلة أونكوميلانيا هوبينسيس للقواقع، والسيطرة على انتقال البلهارسيا في الصين في عام 2015. ولذلك اقترحت خارطة الطريق على مرحلتين للقضاء على البلهارسيا في عام 2015، وذلك بهدف تحقيق انقطاع في انتقال المرض بحلول عام 2020 والوصول إلى التخلص منه بحلول عام 2025 في البلاد. وضعت خلال العقدين الماضيين مجموعة متنوعة من المقاربات، التي تستهدف العوامل الوبائية لمرض الجابونيكا البلهارسيا من أجل منع دورة انتقال الطفيل. وقد استخدمت هذه المقاربات في الأنشطة الوطنية أو المحلية لمكافحة البلهارسيا، وسهّل جزئياً على الأقل عملية تطور برامج القضاء على البلهارسيا. هنا، نقدم مقاربة للسيطرة على مصدر البلهارسيات اليابانية العدوى، ثلاث أدوات جديدة للسيطرة على القواقع، ثلاث مقاربات لكشف ورصد البلهارسيات اليابانية العدوى، ونموذج جديد للتثقيف الصحي. من المتوقع أن تلعب هذه المقاربات دوراً كبيراً في مرحلة التحرك نحو انقطاع انتقال المرض والقضاء على البلهارسيا في الصين.

Translated from English version into Arabic by sjaatoul, through

TRANSLATORS
WITHOUT BORDERS

中国血吸虫病消除规划适宜技术研究进展

孙乐平，汪伟，洪青标，李石柱，梁幼生，羊海涛，周晓农

摘要：

经过 60 余年的积极防治，特别是 2004 年以来以传染源控制为主的血吸虫病综合防治策略的有效实施，中国血吸虫病疫情大幅度降低，并于 2015 年实现了血吸虫病传播控制目标。虽然中国血吸虫病防治工作取得了举世瞩目的巨大成就，但由于日本血吸虫病流行环节多、对人体损害大，其仍然是严重危害居民健康、阻碍社会经济发展的重要公共卫生问题之一。2015 年，中国政府提出了消除血吸虫病分“两步走”的战略目标，即在 2020 年实现血吸虫病传播阻断，到 2025 年消除血吸虫病。在过往的 20 年中，中国血吸虫病防治科研工作者根据日本血吸虫病的流行特征，开发了多种旨在阻断日本血吸虫传播链的适宜技术。这些技术已广泛应用于血吸虫病防治现场实践，并推进了中国消除血吸虫病的防治进程。本文主要介绍了一种渔船民传染源控制技术、三种钉螺控制技术、三种日本血吸虫感染检测和监测技术以及一种健康教育干预新模式。这些防治适宜技术在中国迈向血吸虫病传播阻断和消除进程中将发挥重要作用。

Translated from English version into Chinese by Wei Wang

Examen des approches utilisées dans le programme d'élimination de la schistosomiase en Chine

Le-Ping Sun, Wei Wang, Qing-Biao Hong, Shi-Zhu Li, You-Sheng Liang, Hai-Tao Yang, Xiao-Nong Zhou

Résumé

La schistosomiase japonicum, causée par la schistosome humain *Schistosoma japonicum*, reste un problème majeur de santé publique en Chine, malgré les excellents résultats déjà obtenus. Les efforts destinés à contrôler cette maldie lors des cinq dernières années, notamment le déploiement de la nouvelle stratégie intégrée mettant l'accent sur le contrôle de la source de la schistosomiase japonicum à travers le pays depuis 2004, a considérablement réduit l'apparition de cette chez l'homme, le bétail et son hôte intermédiaire, le mollusque gastropode *Oncomelania hupensis*. D'autre part, le contrôle de la transmission de la schistosomiase a été atteint en Chine en 2015. Une feuille de route en deux étapes a donc été proposée pour l'élimination de la schistosomiase en 2015 visant l'interruption de la transmission de la maladie à l'échelle nationale d'ici 2020 et son élimination d'ici 2025. Au cours des deux dernières décennies, diverses approches visant les facteurs épidémiologiques de la schistosomiase japonica ont été développés en vue rompre le cycle de transmission du parasite. Ces approches, qui ont été adoptées dans le cadre des activités nationales ou locales de lutte contre la schistosomiase, ont permis, au moins en partie, de faire avancer les programmes d'élimination de la schistosomiase. Ici, nous présentons une approche visant le contrôle de la source de la *S. japonicum*, à savoir trois nouveaux outils de lutte contre les mollusques, trois approches de détection et de surveillance de la *S. japonicum* et un nouveau modèle d'éducation sanitaire. Ces approches sont réputées jouer un rôle important dans les efforts déployés visant l'interruption de la transmission de la maladie et l'élimination de la schistosomiase en Chine.

Translated from English version into French by Sandrine Savarit, through

TRANSLATORS
WITHOUT BORDERS

Подходы, используемые в рамках национальной программы ликвидации шистосомоза в Китае: обзор

Ле-Пин Сун, Вэй Ван, Цин-Бяо Хун, Ши-Чжу Ли, Ю-Шэн Лян, Хай-Тао Ян, Сяо-Нонг Чжоу

Реферат статьи

Несмотря на достигнутые успехи, вызываемое шистосомой (*Schistosoma japonicum*) заболевание, известное как японский шистосомоз, остается серьезной проблемой для общественного здравоохранения КНР. Предпринятые в течение последних пяти лет усилия, направленные на контроль над заболеванием, в частности, широкомасштабное внедрение с 2004 года нового комплексного подхода с упором на контроль над источником инфекции *S. japonicum* по всей стране, привели к значительному снижению количества переносчиков *S. japonicum* среди людей,

домашнего скота и промежуточного хозяина, моллюсков рода Онкомелания (*Oncomelania hupensis*). Контроль над передачей шистосомоза был достигнут в КНР в 2015 году. Для полной ликвидации шистосомоза в 2015 году была предложена двухэтапная дорожная карта. Ее целью является достижение прекращения передачи заболевания к 2020 году и победы над ним к 2025 году на всей территории страны. Для того, чтобы блокировать цикл передачи паразита, в течение последних 20 лет было разработано множество подходов, направленных на эпидемиологические факторы японского шистосомоза. Данные подходы использовались в ходе мероприятий по по борьбе с шистосомозом как на национальном, так и на региональном уровнях. Это способствовало, по крайней мере частично, ходу реализации программ по ликвидации шистосомоза. В настоящей работе мы представляем один из подходов к контролю над источником инфекции *S. japonicum*, три новых инструмента для контроля над моллюсками, три подхода к обнаружению и наблюдению за инфекцией *S. japonicum* инфекции, а также перспективную модель обучения в области здравоохранения. Считается, что данные подходы играют большую роль в постепенном прекращении передачи шистосомоза и победе над ним на территории КНР.

Translated from English version into Russian by datran, through

TRANSLATORS
WITHOUT BORDERS

Enfoques aplicados al programa nacional de erradicación de la esquistosomiasis en China: revisión

Le Ping-Sun, Wei Wang, Qing-Biao Hong, Shi-Zhu Li, Te-Sheng Liang, Hai-Tao Yang, Xiao-Nong Zhou

Resumen

La esquistosomiasis japonica, causada por las duelas de la sangre humana del género *Schistosoma japonicum*, sigue siendo un gran problema de salud pública en China, aunque se han registrado importantes avances. Los esfuerzos por controlarla realizados durante los últimos cinco años, en particular la amplia aplicación de la nueva estrategia integrada centrada en el control del origen de la infección por *S. japonicum* en todo el país desde 2004, ha reducido en gran medida la presencia de *S. japonicum* en seres humanos, ganado y *Oncomelania hupensis*, el molusco gasterópodo que le sirve de huésped, y en 2015 se logró controlar la transmisión de la esquistosomiasis en China. Así pues, en 2015 se propuso un plan de trabajo para acabar con la esquistosomiasis, dividido en dos etapas , con el objetivo de interrumpir la transmisión de aquí a 2020 y erradicar la enfermedad en todo el país a más tardar en 2025. Durante las dos últimas décadas, se han desarrollado varios enfoques centrados en los factores epidemiológicos de la esquistosomiasis japonica, con el fin de detener el ciclo de transmisión del parásito. Estos enfoques se han aplicado en las actividades nacionales y locales de control de la esquistosomiasis y han contribuido, al menos en parte, al progreso de los programas de erradicación de la esquistosomiasis. A continuación, presentamos un método para controlar el origen de la infección

causada por *S. japonicum*, tres nuevas herramientas para el control de los moluscos gasterópodos, tres enfoques para la detección y el seguimiento de la infección por *S. japonicum* y un modelo innovador de educación para la salud. Estos enfoques desempeñan un importante papel en el camino hacia la interrupción de la transmisión y la erradicación de la esquistosomiasis en China.

Translated from English version into Spanish by Rvazqg, through

TRANSLATORS
WITHOUT BORDERS
